# Performance on emotional tasks engaging cognitive control depends on emotional intelligence abilities: an ERP study

**DOI:** 10.1038/s41598-017-16657-y

**Published:** 2017-11-27

**Authors:** A. Megías, M. J. Gutiérrez-Cobo, R. Gómez-Leal, R. Cabello, P. Fernández-Berrocal

**Affiliations:** 1 Department of Basic Psychology, Faculty of Psychology, University of Málaga, Málaga, Spain; 2 Department of Developmental and Educational Psychology, University of Granada, Granada, Spain

## Abstract

Cognitive control is a key process in decision making and adequately adapting our behavior to the environment. Previous studies have provided evidence of a lower capacity for cognitive control in emotion-laden contexts in comparison with neutral contexts. The aim of the present research was to study changes in cognitive control performance as a function of emotional intelligence (EI) level in contexts involving emotional information. The study sample was composed of 2 groups of 22 participants each: the high and low EI group. Participants carried out an emotional go/no-go task while brain activity was recorded by EEG. N2 and P3 ERPs were used as indices of cognitive control processing. Participants with higher EI showed a larger N2, reflecting a greater capacity for cognitive control related to changes in conflict monitoring, and to a better detection and evaluation of the emotional stimuli. Moreover, in general, response inhibition accuracy was reduced when emotional information was involved in this process. Our findings reveal that neural mechanisms underlying tasks that engage cognitive control depend on emotional content and EI level. This study indicates the important role played by EI in the relationship between emotion and cognition. EI training may be a very useful tool for improving performance in emotion-laden contexts.

## Introduction

The ability to make decisions in contexts with emotional content is essential in our daily lives. Emotion has an important influence on cognition and vice versa^1,2^. Cognitive processes such as attention, perception, memory, or cognitive control are integrated with emotions so that they jointly contribute to behavior to achieve adequate environmental and social adaptation^3^. The aim of the present research was to study the influence of emotional intelligence (EI) on the relationship between emotional and cognitive processes at both a behavioral and brain level.

EI is a construct that links emotion and cognition, and is defined as the ability to perceive, use, understand, and manage emotions^4^. According to Joseph & Newman^5^ the construct of EI can be categorized into three perspectives depending on the conceptualization and measuring instrument employed: performance-based ability, self-report ability and self-report mixed models. There is increasing evidence in the literature to suggest that the performance-based ability model is the most adequate^6^. This model considers EI as a form of mental ability based on a set of discrete emotional aptitudes which are assessed in an objective manner through performance tests^7^. Gutierrez-Cobo, Cabello, & Fernández-Berrocal^6^, through a systematic review of the literature studying the influence of EI on cognitive processes, showed that when EI is understood as a performance-based ability, it is positively correlated with the performance level in cognitive tasks involving emotional content (hot task). However, this relationship did not exist with cognitive tasks without emotional content (cool task). Thus, higher emotional abilities appear to improve cognitive processing when emotional information is involved in the context.

Among the cognitive processes related to EI, cognitive control has attracted great interest due to its involvement in many aspects of decision making and behavior in general. Cognitive control is a broad construct that refers to goal-directed and self-regulatory processes that allow flexibility in information processing and behavior to vary adaptively as a function of our needs^8,9^. Cognitive control processes include, among others, the ability to selectively retrieve and maintain information related to the task goal, update relevant information during changing environmental conditions, and monitor performance and potential conflicts or inhibition of inappropriate thoughts and responses^10,11^. These abilities are fundamental to our daily activities, allowing us to control our behavior. Deficits in cognitive control lead to problems related to risk behavior, addictions, and aggressiveness^12,13^. Interestingly, many of these contexts are often emotionally charged or at least involve processing of emotional information^14,15^.

Previous studies have provided evidence of a lower capacity for cognitive control in emotional contexts than in those without emotional information^16–18^. For example, Tottenham, Hare, & Casey^17^, using an emotional go/no-go task (a task frequently used to measure the ability to inhibit a prepotent response, an important aspect of cognitive control), found that emotional information interferes with cognitive control by showing a higher false alarm rate to emotional no-go stimuli than to neutral stimuli. In a similar vein, Gutierrez-Cobo, Cabello, & Fernández-Berrocal^18^ went a step further and investigate possible differences in cognitive control as a function of emotional abilities. Individuals with higher EI levels presented greater cognitive control (less false alarms) in an emotional go/no-go task compared with those who have lower EI levels. Interestingly, both groups (high and low EI) performed equally in the go/no-go task when the task did not involve emotional information. In light of these results, and given the large number of emotion-laden contexts we face each day and the importance of cognitive control in our behavior, there is clearly great value in having adequate emotional intelligence abilities for adapting to the environment.

An additional and useful measure for assessing cognitive control are event-related brain potentials (ERPs)^19–21^. Previous literature using ERPs in go/no-go tasks has linked cognitive control with two main ERPs components that are specifically related to the processing of no-go trials: N2 and frontal P3^22,23^. The N2 component is a negative deflection over fronto-central electrodes peaking between 200 and 350 ms after stimulus onset, and is maximal in frontal areas. N2 is interpreted as representing early processes involved in response inhibition, particularly conflict monitoring between competing responses^9,24^. The characteristic frontal P3 usually found in go/no-go task is a positive wave peaking at fronto-central electrodes between 300 and 500 ms post-stimulus onset. It is interpreted as a response inhibition index associated with the cognitive and motor processes implied in the cancellation of the anticipated response^22,24,25^. N2 and P3 both increase with higher cognitive control demand. Therefore, in a go/no-go task, both components should show larger amplitudes for no-go stimuli relative to go stimuli^23^.

These brain electrophysiological correlates could be an excellent instrument to investigate the influence of EI on cognitive control processes. However, to date, and as previously described, evidence has only been shown at the behavioral level^17,18^. Therefore, exploring and clarifying this issue could help to make a significant contribution to the understanding of the emotion and cognition integration mechanisms.

The aim of the present research was to study the electrophysiological basis of the changes in cognitive control performance as a function of EI level in tasks or contexts involving emotional content. To carry out the study, we used an emotional go/no-go task, a paradigm that has been well-established in previous literature^18,23,26^. Following similar methods to those shown in previous work, changes in frontal N2 and P3 ERP amplitudes were assessed as indices of cognitive control processing^22,27,28^. We hypothesized that individuals with high EI will show a greater level of cognitive control in the emotional go/no-go task than individuals with low EI, which must be reflected in a higher accuracy, and a larger N2 and P3 amplitude on no-go trials. In addition, and on the basis of previous results in the literature, differences between go and no-go trials should be evidenced by lower accuracy and larger N2 and P3 for no-go trials in both EI groups.

## Results

Four participants were excluded from the study due to poor EEG recording quality or excessive movement artifacts. The final sample included 40 participants, 21 in the high EI group and 19 in the low EI group.

### Behavioral results

The Group x Stimulus Type x Emotion ANOVA conducted on the accuracy scores revealed a main effect of Stimulus Type, *F* (1, 38) = 44.76, MSE = 0.16, *p* < 0.001, η^2^_p_ = 0.54, Emotion, *F* (2, 76) = 6.84, MSE = 0.03, *p* < 0.01, η^2^_p_ = 0.15, and the interaction Stimulus Type x Emotion, *F* (2, 76) = 19.29, MSE = 0.002, *p* < 0.001, η^2^_p_ = 0.34. Post-hoc analysis of the interaction revealed a higher accuracy on go trials than on no-go trials for the three types of emotional faces (all p < 0.05). Moreover, on go trials, happy faces had a higher accuracy than neutral and fear faces (M_go-neutral_ = 0.95; M_go-fear_ = 0.95; M_go-happy_ = 0.97; all *p* < 0.05). And, on no-go trials, neutral faces had a higher accuracy than fear and happy faces (M_nogo-neutral_ = 0.89; M_nogo-fear_ = 0.84; M_nogo-happy_ = 0.82; all *p* < 0.05).

The Group x Emotion ANOVA conducted on reaction times revealed a main effect of Emotion *F* (2, 76) = 13.74, MSE = 1265.39, *p* < 0.001, η^2^_p_ = 0.27. Participants responded faster to happy faces than to neutral and fear faces (M_neutral_ = 486 ms; M_fear_ = 485 ms; M_happy_ = 450 ms; all *p* < 0.05).

In addition, due to the long duration of the task, we decided to study the possible effects of fatigue on task performance. We conducted two separate ANOVAs on the accuracy and reaction time data with Blocks as the independent variable. The analyses did not reveal any main effects of Block (accuracy: *F* (5, 195) = 0.86, MSE = 0.002, *p* = 0.51; reaction times: *F* (5, 195) = 1.65, MSE = 1437.23, *p* = 0.15).

### ERP results

**Figure 1:**
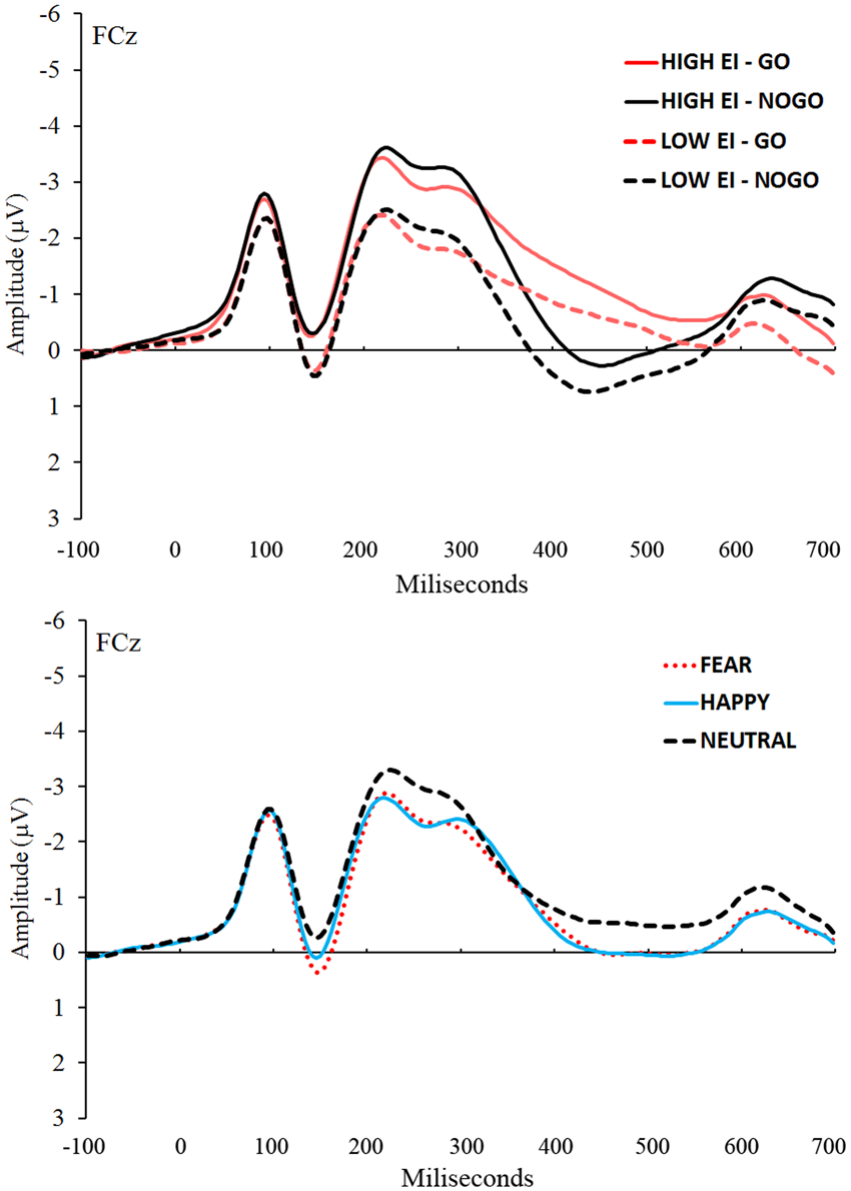
Top panel: Grand average waveforms for each Group and Stimulus Type condition at FCz in the −100 to 700 ms interval time-locked to the face stimulus onset. Bottom panel: Grand average waveforms for each Emotion condition at FCz in the −100 to 700 ms interval time-locked to the face stimulus onset.

The Group x Stimulus Type x Emotion ANOVA conducted on the average N2 amplitude revealed main effects of Group, *F* (1, 38) = 4.19, MSE = 20.92, *p* = 0.04, η^2^_p_ = 0.10, Stimulus Type, *F* (1, 38) = 4.57, MSE = 0.75, *p* = 0.03, η^2^_p_ = 0.11, and Emotion, *F* (2, 76) = 11.92, MSE = 0.43, *p* < 0.001, η^2^_p_ = 0.24. No interactions were significant. Figure 1 depicts the grand average waveforms for Group and Stimulus Type conditions (top panel) and for the Emotion conditions (bottom panel).

**Figure 2:**
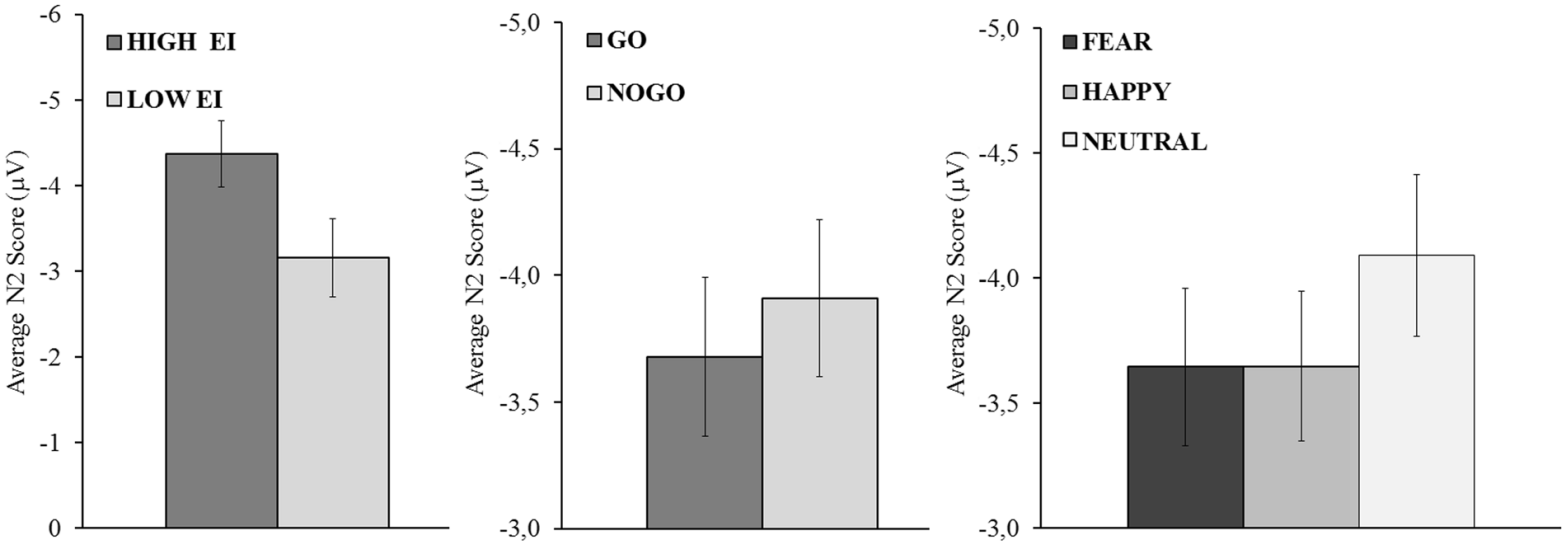
Average N2 amplitude at FCz for each level of Group, Stimulus Type and Emotion independent variables. The Y-axis was inverted in order to facilitate the understanding of the N2 differences. Vertical bars represent the standard error of the mean.

The High EI group showed a larger N2 than the low EI group (M_highEI_ = −4.37; M_lowEI_ = −3.16; *p* < 0.05). No-Go trials showed a larger N2 than go trials (M_nogo_ = −3.91; M_go_ = −3.68; *p* < 0.05) and neutral faces showed a larger N2 than fear and happy emotional faces (M_neutral_ = −4.09; M_fear_ = −3.65; M_happy_ = −3.65; all *p* < 0.05, Bonferroni corrected). Figure 2 displays average N2 amplitudes for each significant main effect.

**Figure 3:**
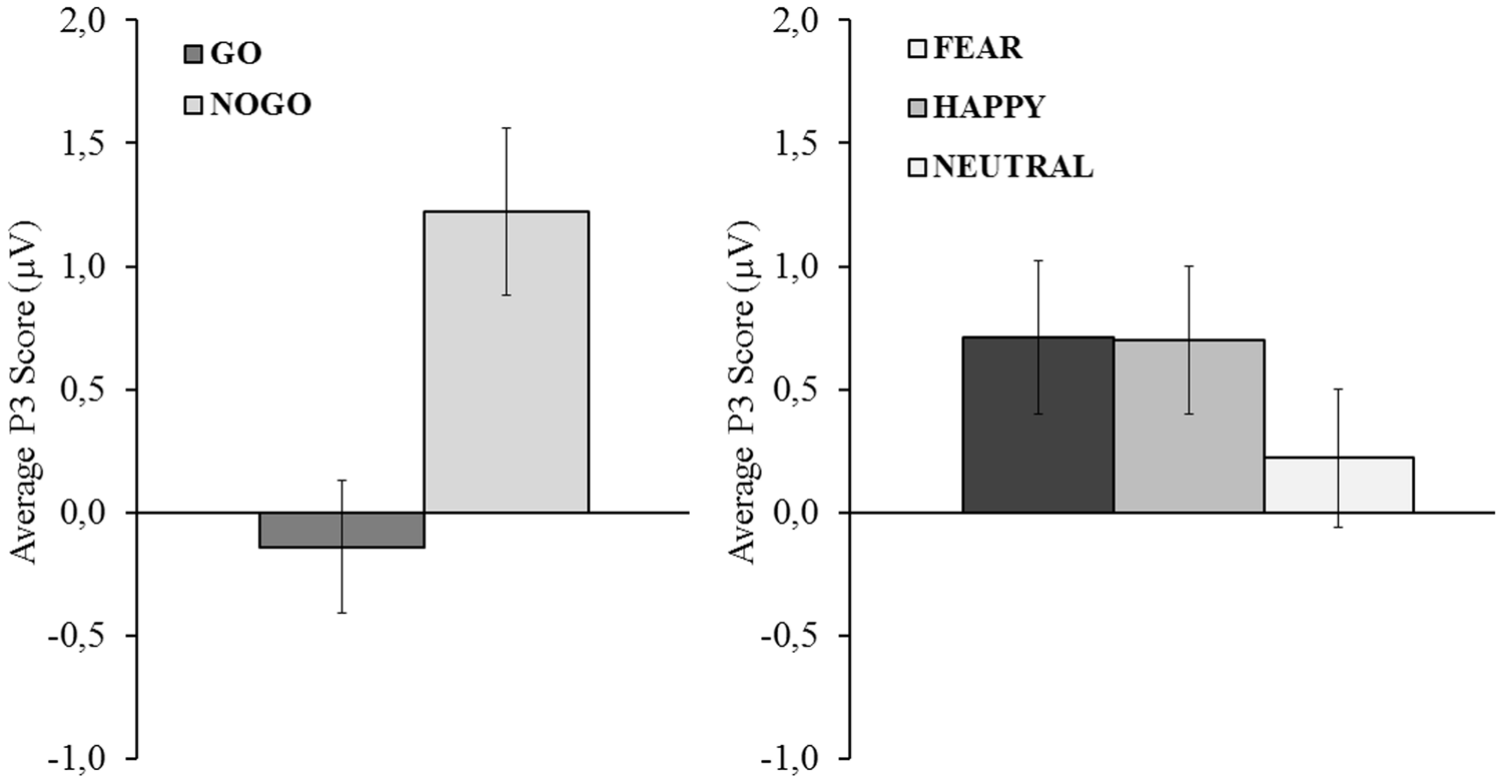
Average P3 amplitude at FCz for each level of the Stimulus Type and Emotion independent variables. The vertical bars represent the standard error of the mean.

With respect to the P3 component, The Group x Stimulus Type x Emotion ANOVA revealed a main effect of Stimulus Type, *F* (1, 38) = 46.59, MSE = 2.39, *p* < 0.01, η^2^_p_ = 0.55, and Emotion, *F* (2, 76) = 8.98, MSE = 0.69, *p* = 0.03, η^2^_p_ = 0.19. No-Go trials showed a larger P3 amplitude than go trials (M_nogo_ = 1.27; M_go_ = −0.14; *p* < 0.05). Neutral faces showed a reduced P3 amplitude compared with fear and happy emotional faces (M_neutral_ = 0.27; M_fear_ = 0.71; M_happy_ = 0.70; *p* < 0.05, Bonferroni corrected). Figure 3 displays average P3 amplitudes for each significant main effect.

Additionally, given that potential amplitude differences between EI groups were visible on the grand average ERP waveforms prior to N2 (see Fig. 1), we decided to analyze the positive potential deflection occurring around 150 ms after face stimulus onset, in order to discard any influence of other ERPs on N2 and P3 differences. The P150 component was computed as the mean amplitude of the +−12 ms period around the most positive peak in the 100–250 ms interval after face stimulus onset. A t-test with Group as the independent variable revealed no significant differences between high and low EI groups (*p* > 0.05).

## Discussion

The main objective of the present research was to further advance existing knowledge of the relationship between emotion and cognitive control processes. In particular, we were interested in exploring how cognitive control ability and its underlying electrophysiological mechanisms depend on individual EI levels when performing tasks that include emotional content. We explored this issue through a well-established emotional go/no-go task which has been widely used in previous research to assess cognitive control and its interaction with emotional information^6,17,23^. The behavioral task was accompanied by an EEG recording in order to determine brain activity changes related to cognitive control.

Firstly, we verified that the go/no-go task worked properly in accordance with previous research^17,23^. As expected, no-go trials showed a lower accuracy than go trials. The higher frequency of go stimuli increased the difficulty of response inhibition on no-go trials and, thus, the likelihood of making errors. These behavioral differences should be reflected in the EEG recording by changes in N2 and P3, two indices of cognitive control performance^23^. In our study, larger N2 and P3 were evident on no-go trials compared with go trials, which indicates increased cognitive control. Thus, the behavioral and electrophysiological results were in accord with those of previous studies using the go/no-go paradigm.

Secondly, having verified the correct functioning of the go/no-go task, and to achieve the main objective of the present study, we focused on differences in cognitive control as a function of EI level. Participants with high EI showed a larger N2 than those with low EI. This finding is in line with previous behavioral research showing that individuals with higher EI possess a greater capacity for cognitive control during the performance of tasks involving emotional content^29^. Importantly, in our study, the main effect of the EI group was observed on both go and no-go trials. Following our initial hypothesis, we expected to find differences between groups in the N2 component only on no-go trials, or at least larger differences compared with go trials, given their greater involvement in cognitive control processes. The differences on no-go trials may be explained by a greater cognitive control capacity of the high EI group during the execution of an emotional go/no-go task, however, although go trials also require cognitive control^20^, comparable differences between go and no-go trials do not seem to fit well with this explanation.

N2 results similar to those found in our study have also been observed in previous studies in the literature comparing cognitive control abilities between different sample populations^30–34^. For instance, recent studies, such as those of Cid-Fernández *et al*.^31^ or Mäki-Marttunen^33^ have shown a lower N2 amplitude responding to both go and no-go trials in patients with mild brain damage affecting cognitive control. These differences at brain level are also supported by behavioural studies showing that poorer performance on no-go trials is often accompanied by poor performance on go trials (for example in gamblers^35^, smokers^36,37^, adults with attention deficit hyperactivity disorder^38^, or adolescents facing alcohol cues^39^). The N2 component on go trials has usually been associated with allocation of resources to the detection and categorization of the target stimulus, and its evaluation and comparison in working memory^30,31,34^. Therefore, the N2 differences between groups may reflect deficits in the mobilization of these resources. In our study, the stimuli that the participants had to detect and evaluate were faces with emotional expressions. People with high emotional intelligence show better recognition of emotional faces^40^. Thus, it seems logical to think that N2 differences between EI groups on go trials may be explained by differences in the processing of the presented stimuli. This idea could also be applied to N2 differences on the no-go stimuli since, although the literature has shown that this component is strongly associated with cognitive control *per se*, better stimulus processing may also lead to superior cognitive control performance.

In summary, previous research has shown a deficit in cognitive control performance in emotion-laden contexts compared with neutral contexts^17,18^. Our results suggest that people with higher EI abilities may reduce this deficit due to a greater cognitive control capacity as well as better target stimulus detection and evaluation. Further studies are needed in order to adequately dissociate both effects.

On the other hand, no differences were observed between EI groups in terms of P3 amplitude. According to our hypothesis, we expected to find significantly larger P3 amplitude for the high EI group on no-go trials (compared with the low EI group). Although both N2 and P3 ERP components are considered to be indices of cognitive control in the processing of no-go trials, there is evidence that the N2 and P3 ERPs represent different phases over the time course of cognitive control mechanisms^23,41^. P3 reflects inhibitory processing associated with the cancellation of an anticipated response^42^, while N2 appears to be more associated with a previous step, the conflict detection between two or more representations of incompatible responses, in our case, between the planned response and the demanded response^20,21^. It is not possible for response inhibition to occur without a prior response conflict^41^. Thus, based on our N2 results, the differences found between the EI groups on no-go trials could be explained by changes in conflict monitoring processes rather than response inhibition.

Finally, we also found significant differences related to the emotional face conditions. The results revealed higher accuracy and lower reaction times on happy faces than on fear and neutral faces for go trials, and a higher accuracy on neutral faces than on fear and happy faces for no-go trials. Similar results were found in the study by Tottenham *et al*.^17^, showing that participants are more accurate when making positive discrimination (go stimulus) of happy faces. However, they showed a poorer accuracy rate for emotional no-go stimuli than neutral no-go stimuli suggesting that the presence of emotional information may interrupt regulatory control of impulse behavior. With respect to the ERP results, neutral faces showed a more negative N2 and P3 amplitude than Fear and Happy emotional faces. This finding is consistent with the study by Zhang & Lu^23^. These authors explain differences in N2 in terms of top-down attention toward emotions. In particular, attention is automatically focused on emotional faces^43^, resulting in a lower need to allocate top-down attentional resources to the goal-directed behavior, which is reflected in a smaller N2. Subsequently, these resources may be available to improve emotional regulation processes and carry out a better automatic response inhibition of emotion, which gives rise to a larger P3 in the emotional face stimuli^23^. In any case, further research is needed in order to clarify the specific effect of emotional face valence on go and no-go stimuli, given that although there is support for our results in the literature, there are some studies showing controversial behavioral results^44,45^.

As limitations of the study, it is important note that, although our experimental manipulation showed brain electrophysiological differences between high and low EI groups, the task was not sensitive to behavioral changes. Based on prior research^18^, we expected that higher EI participants would show a higher number of false alarms (i.e. worse response inhibition performance on no-go trials) than lower EI participants. Nevertheless, although it is true that the averaging processes in ERP analyses remove much information^46^, these are usually more sensitive to differences in cognitive processes than behavioral analyses^47^. Thus, behavioral changes in our task could require greater differentiation between EI levels. Future studies should work with more extreme populations in EI to address this question. In any case, this issue, far from being an impediment, revealed that ERPs may be used as an excellent and sensitive assessment tool for the study of changes in cognitive control demand and the influence of EI on cognitive processes. It would be of interest to investigate the effect of EI abilities on other cognitive processes such as memory, attention or learning by ERP techniques. Additionally, further research with gender-matched samples would allow for exploring possible gender differences. In our study, although the distribution of gender was similar in high and low groups, there were an unequal number of men and women in the total sample, which prevented us from conducting this type of analysis. Finally, the use of neuroimaging techniques such as fMRI or PET could provide new and interesting information about the brain areas implicated in the integration of emotion and cognitive control as a function of EI.

Our results also have clinical implications. Future research should focus on how EI training may benefit individuals’ cognitive control ability. Given that deficits in this cognitive ability are related, for instance, to impulsiveness, risk behavior, drug abuse or over-consumption of caffeine^13,48–51^, finding new procedures for reducing these deficits could have remarkable consequences. EI training has already shown to be an effective tool for improving other relevant variables such as aggression, empathy or mental health in adolescents^52–54^. Therefore, our findings open up a promising new line of intervention in order to discover if EI training could enhance the capacity for cognitive control in emotional contexts, given the relations found in the present study.

To summarize, cognitive control is a key process in decision-making and adapting our behavior to the environment. The present study supports the notion that the capacity for cognitive control is impaired when emotional information is involved in cognitive tasks, reducing the ability to regulate behavior. In addition, and as a main objective, it was found that electrophysiological mechanisms underlying the execution of tasks that involve cognitive control depended on emotional abilities. Individuals with higher EI presented a greater capacity for cognitive control, reflected in a larger N2 ERP component on no-go trials. Moreover, between group differences on N2 on go trials were associated with performance changes in the detection and evaluation of the target stimuli. These findings constitute further evidence of the strong integration of emotion and cognition, and the potentially important role for EI in linking both constructs. From an applied point of view, our results suggest that training in EI abilities may be very beneficial for adequate performance in emotional contexts.

## Methods

### Participants

Participants in this study were selected from a pool of 209 students at the University of Málaga who had been previously assessed for EI by the Mayer-Salovey-Caruso Emotional Intelligence Test (MSCEIT)^55,56^. Individual scores from this scale were used to divide the study sample into two groups: High EI and Low EI. Participants belonging to the high and low EI group were chosen from the 25% highest and lowest EI scores, respectively.

The sample of participants included in the experiment was composed of 44 volunteer participants (22 in the high EI group and 22 in the low EI group). There were 40 women and 4 men. The average age was 22.16 years (SD = 4.95). The distribution of gender and age was similar in both groups (Low EI: 20 women and 2 men, average age = 22.85; High EI: 20 women and 2 men, average age = 21.5). An independent samples t-test on age between EI groups showed no significant differences (*p* = 0.59). All participants took part in the experiment in exchange for course credits, gave signed, informed consent and were treated in accordance with the Declaration of Helsinki^57^. The Research Ethics Committee of the University of Málaga approved the study protocol as part of the projects SEJ-07325 and PSI2012-37490.

### Instruments and Procedure

The Mayer-Salovey-Caruso Emotional Intelligence Test (MSCEIT)^56^ is a performance-based ability measure of EI. The MSCEIT is composed of 141 items divided in four branches according to Mayer and Salovey’s theory^58^: perceiving, facilitating, understanding, and managing emotions. The instrument provides separate scores for each branch and an overall EI score. In our study, EI abilities were measured using a Spanish version of MSCEIT, which shows adequate psychometric properties similar to the English original version (reliability index for expert criterion is *r* = 0.93 and for general criterion *r* = 0.94)^55^.

Having selected the participants belonging to the High and Low EI by MSCEIT scores, the go/no-go task was conducted, along with EEG recording. Participants performed the experimental task in a quiet room on a 21-inch monitor at a resolution of 1200 × 800 pixels placed approximately 60 cm away from them. The task was developed and run in E-Prime software (Psychology Software Tools Inc., Pittsburgh, USA). Two computers running under Windows 7 with Intel Core 2 Duo processors at 2.8 GHz and 4 Gb RAM were used for controlling the task and recording EEG activity.

**Figure 4:**
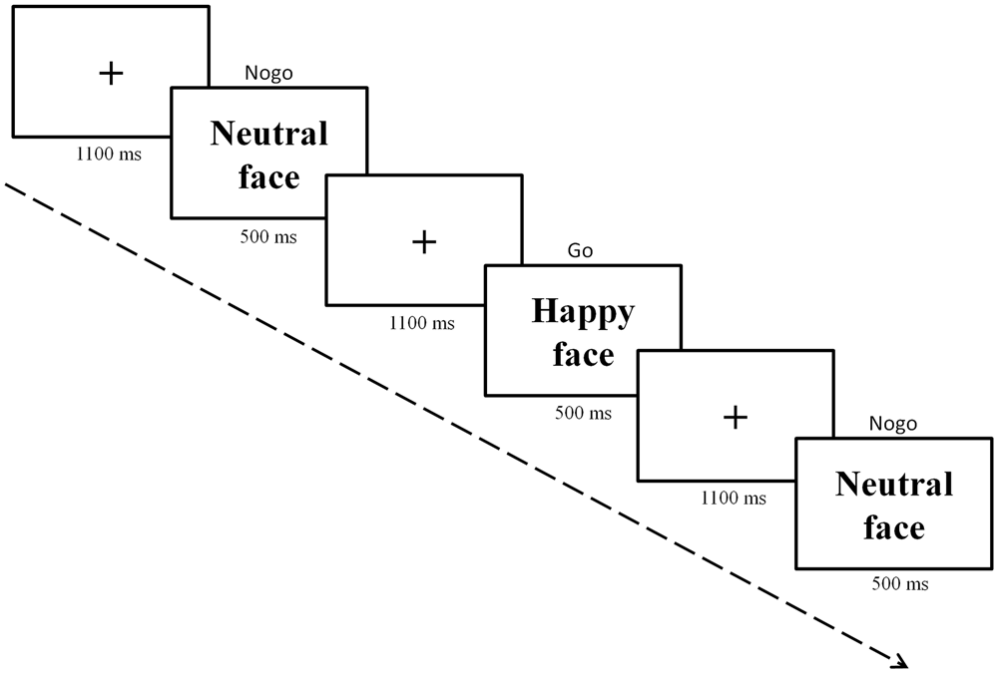
Example of a sequence of trials.

Following a standard go/no-go task paradigm, participants had to respond by pressing the space bar on go trials, and to withhold their response on no-go trials. Go trials were more frequently presented (70%) than no-go trials (30%) to promote the tendency to respond and commit more false alarms. The go and no-go stimuli employed in our task were 13 faces of adult females and males, each of them showing three emotional expressions: happy, fear and neutral (some examples can be seen in Fig. 4). Thus, a total of 39 different faces were used. All of these were extracted from the NimStim set^59^, available at http://www.macbrain.org. The task was composed of 6 blocks of 267 trials (whole task: 1602 trials). Prior to each block, participants were informed that a particular facial expression would be the go stimulus. Each block presented a different combination of go/no-go stimuli and facial expression (go/no-go: fear/happy, fear/neutral, happy/fear, happy/neutral, neutral/fear, neutral/happy). The trial sequence consisted of a fixation point displayed for 1100 ms, after which the face stimulus was presented for 500 ms (see Fig. 4). The order of the blocks and trials within blocks were randomized across participants. Participants were instructed to respond as fast and as accurately as possible. Before starting the task, a short practice session was completed in order to familiarize participants with the task. The whole task lasted approximately 45 minutes (plus the time required for preparation of EEG recording: approximately 35–45 min).

### EEG recording, pre-processing and ERP measures

The EEG signal was recorded using a 32-channel BrainCap system (Brain Products Inc., Germany), in which electrodes are positioned on a nylon cap according to the international 10–20 system^60^. Vertical and horizontal electro-oculograms (VEOG and HEOG) were recorded from two electrodes located on the outer canthus and below the right eye. We always kept electrode impedances below 5 kΩ. The amplified EEG and OEG were sampled at a frequency of 1000 Hz and 0.016–100 Hz band-pass filter. The online reference electrode was the outer canthus of the right eye.

Continuous EEG signals were preprocessed offline using EEGLAB^61^. The raw data were downsampled to 250 Hz, band-pass filtered using a 0.2–40 Hz (12 db/octave), and re-referenced to the average of all electrodes. All channels having longer flatline than 50 s or a correlation index lower than 0.6 with respect to a channel reconstruction based on their neighboring channels were rejected by clean rawdata EEGLAB plug-in^62–64^. The average number of rejected channels per participant was 0.21 (SD = 0.51). Rejected channels were interpolated by spherical spline method^65^.

EEG data were segmented into epochs of 1600ms, from −100 to 1500 ms time-locked to the face stimulus onset. Baseline correction was performed using the average EEG activity in the first 100ms of each epoch. Artifacts were corrected by the Second Order Blind Identification algorithm (SOBI)^66,67^. Epochs with amplitudes exceeding +/−75 mV were excluded from analysis. The mean number of accepted epochs per block for go and no-go conditions was 178.8 (SD = 18.1) and 77.3 (SD = 7.3) respectively. Finally, epochs were averaged separately per participant for each condition of the experimental design (2 [EI: High and Low) × 2 [Type of trial: Go and No-go] × 3 [Emotion: Happy, Fear, and Neutral]].

In accord with previous literature regarding the emotional go/no-go task^23^ and on the basis of the inspection of grand average ERP waveforms, we decided to compute the N2 and P3 components as follows. The N2 component was defined for each participant and condition as the mean amplitude of the +−12 ms period around the most negative peak in the 150–350 ms time after face stimulus onset at the FCz site. The P3 component was defined for each participant and condition as the mean amplitude of the +−12 ms period around the most positive peak in the 300–500 ms time after face stimulus onset at the FCz site. FCz electrode was chosen for both components since the literature has demonstrated that the largest response is at fronto-central sites^23^.

### Statistical analysis

For behavioral analysis, accuracy (probability of a correct response) was submitted to a 2 × 2 × 3 mixed ANOVA with Group (high EI, low EI) as the between-subject variable, and Stimulus Type (go and no-go) and Emotion (happy, fear, and neutral) as within-subject variables. Mean reaction times for correct trials were submitted to a 2 × 3 mixed ANOVA with Group and Emotion as independent variables.

With respect to the ERP analysis, the average amplitudes of the N2 and P3 components were submitted separately to a 2 × 2 × 3 mixed ANOVA with Group (high EI, low EI) as the between-subject variable, and Stimulus Type (go and no-go) and Emotion (happy, fear, and neutral) as within-subject variables.

### Data Availability

The datasets generated during and/or analyzed during the current study are available from the corresponding author on reasonable request.
